# Supplementary material for: Complete Mapping of Mutations to the SARS-CoV-2 Spike Receptor-Binding Domain that Escape Antibody Recognition
Source: Cell Host Microbe. 2021 Jan 13;29(1):44–57.e9. doi: 10.1016/j.chom.2020.11.007 (PMC7676316; doi:10.1016/j.chom.2020.11.007)
Supplement: Document S1. Figures S1–S6 and Table S2 [file mmc1.pdf]

Cell Host & Microbe, Volume *29*

Supplemental Information

Complete Mapping of Mutations to the SARS-CoV-2 Spike Receptor-Binding Domain that Escape Antibody Recognition

Allison J. Greaney, Tyler N. Starr, Pavlo Gilchuk, Seth J. Zost, Elad Binshtein, Andrea N. Loes, Sarah K. Hilton, John Huddleston, Rachel Eguia, Katharine H.D. Crawford, Adam S. Dingens, Rachel S. Nargi, Rachel E. Sutton, Naveenchandra Suryadevara, Paul W. Rothlauf, Zhuoming Liu, Sean P.J. Whelan, Robert H. Carnahan, James E. Crowe Jr., and Jesse D. Bloom

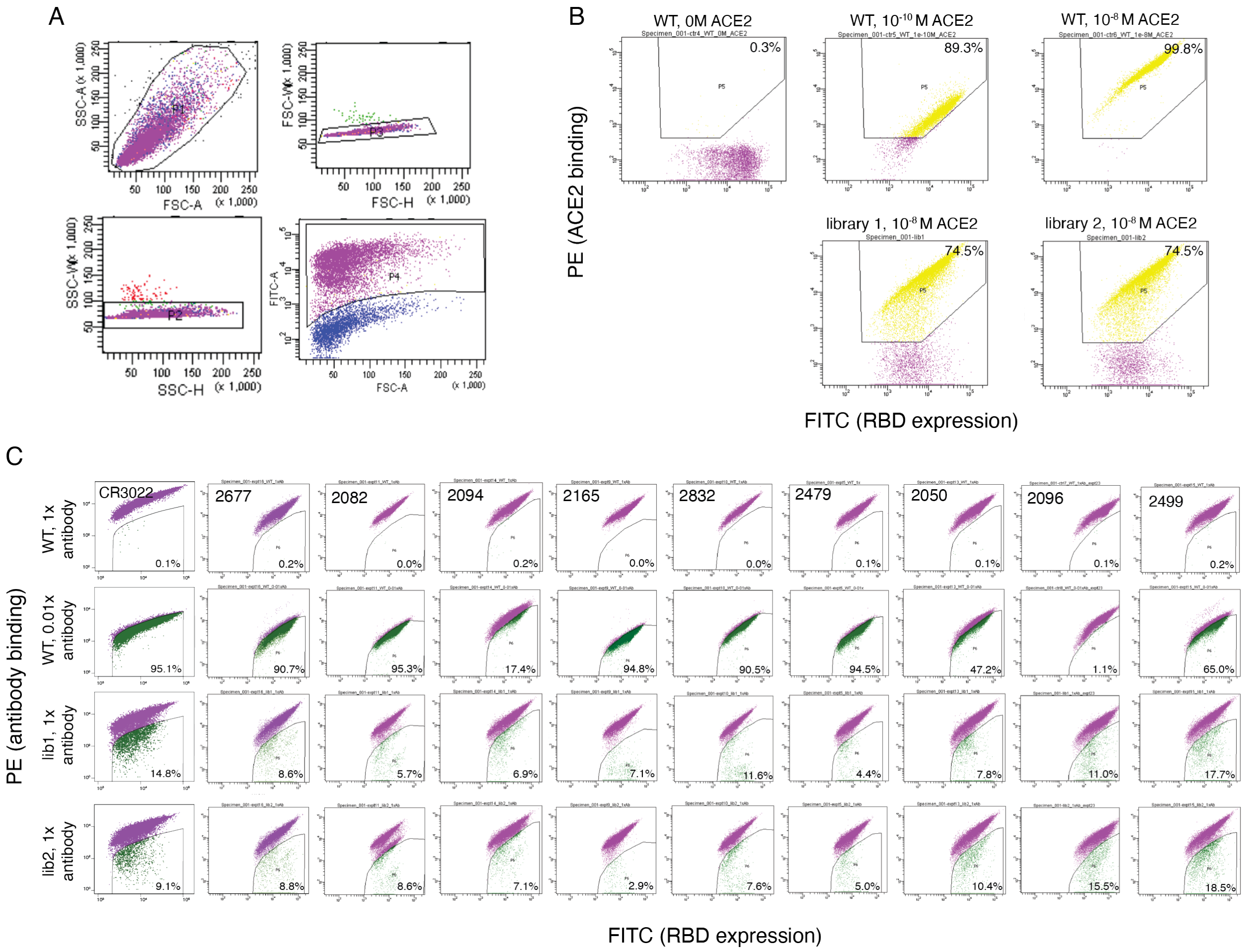

Figure S1. FACS gating, related to Figure 1. (A) Representative hierarchical gates drawn to isolate RBD+ single cells as the parent population for FACS gates in (B, C). First, hierarchical gates were drawn to select single-cell events: forward scatter (FSC) versus side scatter (SSC, top left), SSC width versus height (bottom left), and FSC width versus height (top right). Next, FITC+ labeling of a C-terminal epitope tag on the RBD was used to identify RBD+ cells (purple, bottom right). Selection gates for ACE2+ and antibody-negative sorts (B, C) are nested within this RBD+ population. (B) RBD mutant libraries were first sorted for variants that could bind ACE2 with at least 0.01x the affinity of unmutated SARS-CoV-2 RBD, the approximate affinity of RaTG13, the homolog with the lowest affinity that still marginally mediates cell entry (Shang et al., 2020). Top three plots show unmutated SARS-CoV-2 labeled at 0 M, 1e-10 M, and 1e-8 M ACE2. A selection gate was drawn to capture unmutated cells labeled at 1e-10 M ACE2. The bottom two plots show the application of this selection gate to the duplicate RBD mutant libraries labeled at 1e-8 M ACE2. Percentages of RBD+ cells (yellow) in each control and library sample that fall into the ACE2+ sort bin are shown in the upper-right of each FACS plot. These ACE2+ sorted libraries were grown overnight and used for subsequent antibody-escape selections. (C) Selection gates for the antibody-escape sorts. Unmutated SARS-CoV-2 RBD was labeled at 400 ng/mL (1x) and 4 ng/mL (0.01x) with each antibody. Antibody-escape selection gates were drawn to capture 0.2% or less of the 1x and up to 95% of the 0.01x antibody-labeled unmutated RBD control cells. Each mutant RBD library was labeled with 400 ng/mL (1x) antibody, and cells that were captured in the “antibody-escape bin” were sorted and their barcodes were sequenced. Percentages of RBD+ cells in each control and library sample that fall into the antibody-escape bin are shown in the bottom-right of each FACS plot.

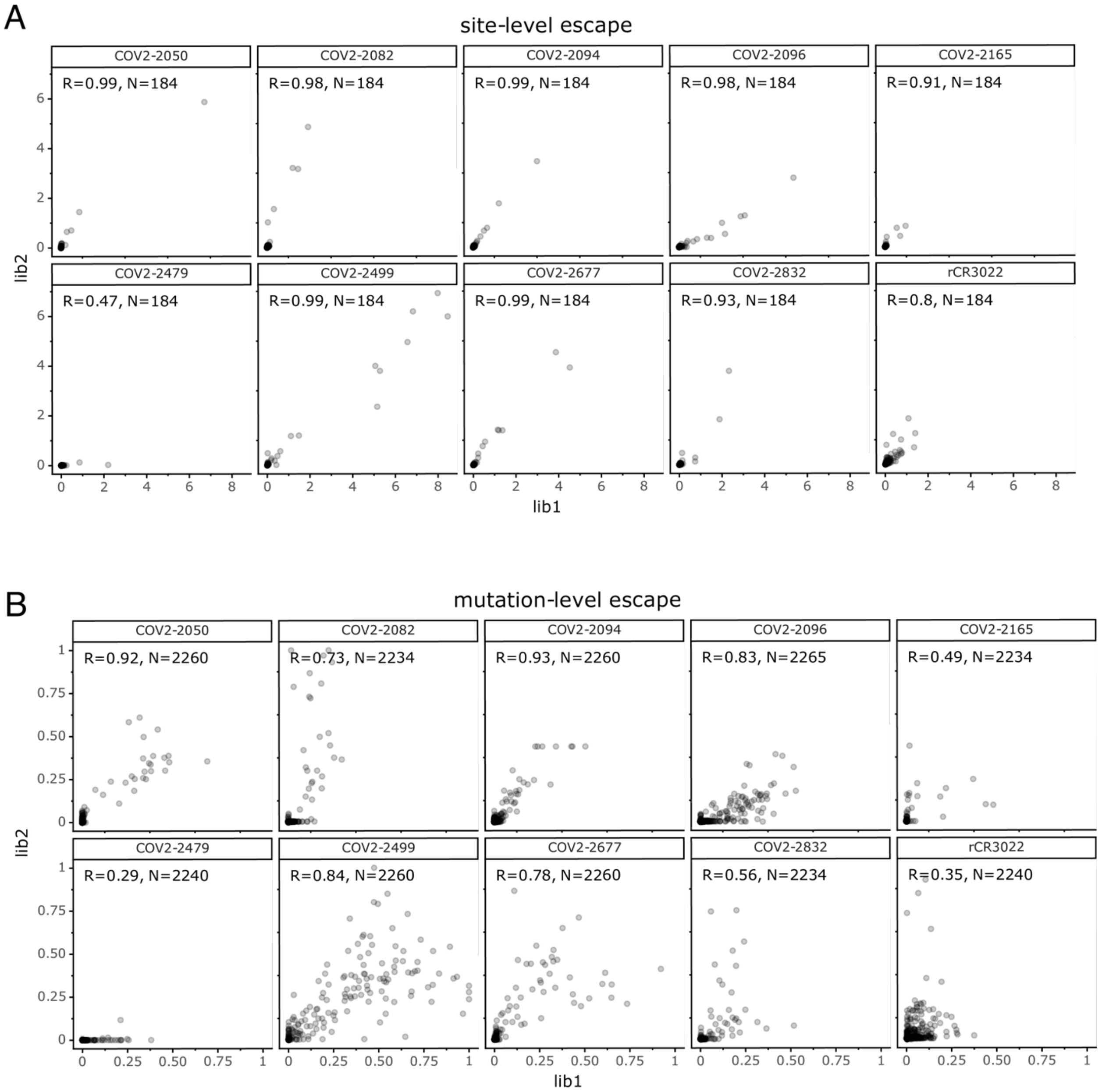

Figure S2. Correlation between the duplicate mappings of escape mutations made with the independently generated mutant virus libraries ("lib1" and "lib2"), related to Figure 2. (A) Correlation between the total escape at each site. (B) Correlation between the escape fraction measured for each individual mutation. The text insets in each plot give the Pearson's correlation coefficient and the number of sites or mutations for which measurements were made for both libraries. The data shown in the rest of the paper are the average of those from the two libraries. Though there is some variation between replicates in the escape fraction of individual mutations (particularly for antibodies with smaller-magnitude escape) (B), correlation in the site-wise sum escape (A) is reasonable for all antibodies.

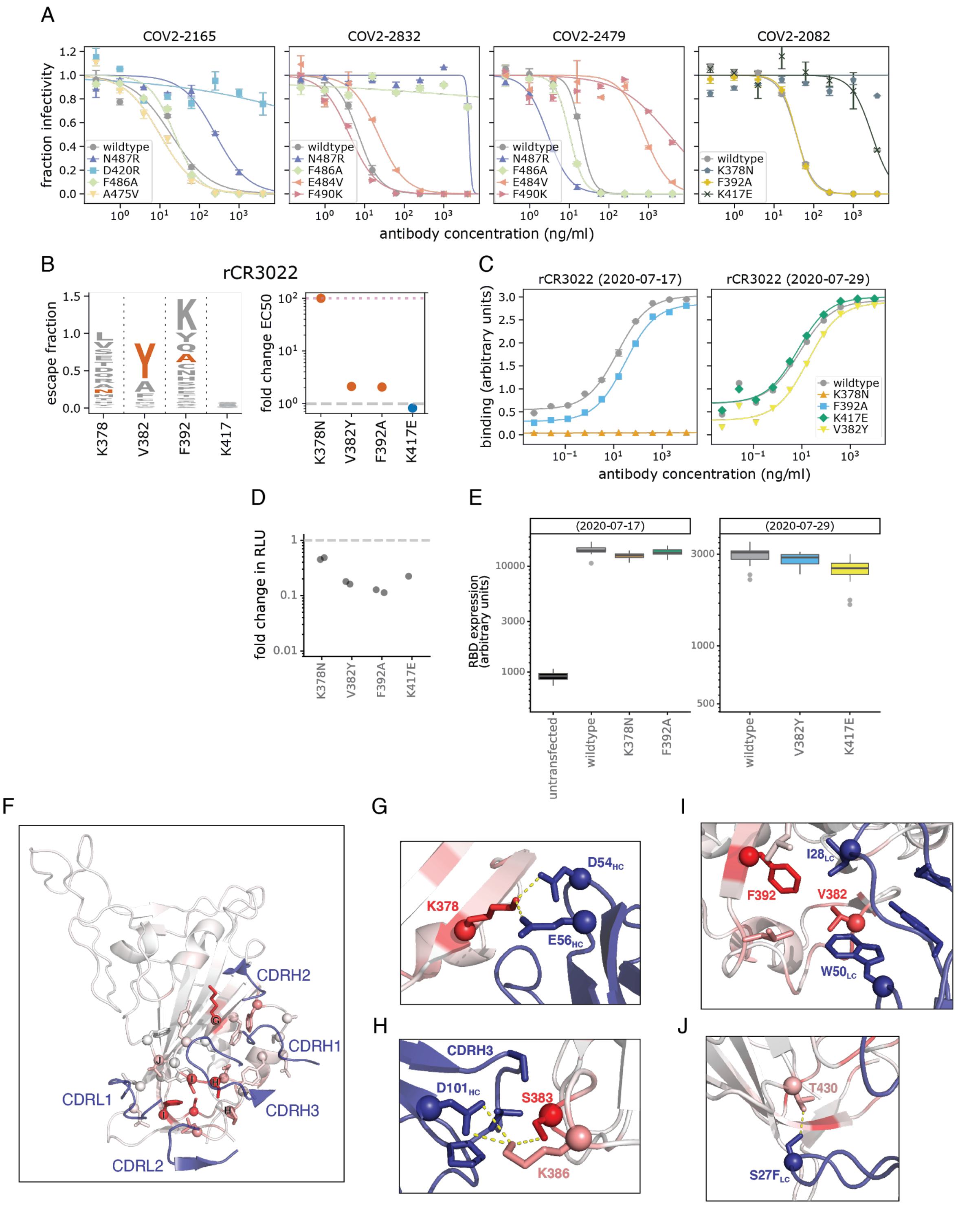

Figure S3. Validation of the functional and structural relevance of antibody-escape maps, related to Figures 3 and 4. (A) Neutralization curves with the spike-pseudotyped lentiviral particles used to determine IC50 values plotted in Figure 3. Each point represents the mean and standard error of 2 independent measurements. The IC50s were computed using the `neutcurve` package (https://jbloomlab.github.io/neutcurve/) to fit two-parameter Hill curves (with the baselines fixed to 0 and 1). IC50s outside the range of tested antibody concentrations are reported as upper bounds. (B) Antibody

rCR3022 is non-neutralizing, so we instead used flow cytometry to measure rCR3022 binding to RBD expressed on the surface of mammalian cells (see Methods for details), with the values representing the fold change in effective concentration 50% (EC50) for antibody binding to each mutant RBD relative to wildtype. (C) The binding curves summarized in (B), with the y-axis representing binding as measured by flow cytometry. EC50s are computed using the `neutcurve` package to fit four-parameter Hill curves (both baselines free) and the midpoint is reported as the EC50. The assays were performed on two separate days, and fold changes are computed relative to the unmutated (wildtype) RBD from that day. (D) rCR3022 escape mutations are compatible with function in spike-pseudotyped lentiviral particles. The infectious titer of spike-pseudotyped lentivirus mutants in transfection supernatants as quantified by fold change in relative luciferase units (RLUs) compared to virus pseudotyped with the unmutated (wildtype) spike. All titers were measured in biological duplicate transfections (two jittered points) except K417E. (E) To estimate RBD expression on the surface of 293T cells in the rCR3022 binding assays in panels B and C, cells were also labeled with biotinylated ACE2 and fluorophore-conjugated streptavidin. ACE2 binding levels, a proxy for RBD expression, were measured by flow cytometry. Box plots represent the median and 25th and 75th percentiles, whiskers are 1.5 * interquartile range, and outliers are shown individually. For each condition, n=12-24. (F-J) Structural analysis of rCR3022 escape in the high-resolution CR3022-bound RBD structure (PDB: 6W41 (Yuan et al., 2020)). (F) Escape at CR3022-contact residues. RBD residues are colored by total site-wise escape, from white (0 total escape) to red (maximum total escape). CR3022 CDR loops that mediate RBD contacts are shown in blue and labeled. Side chains are shown as sticks and C_α spheres are shown for RBD residues defined as CR3022 structural contacts (non-hydrogen atoms within 4 Å distance) or sites of strong selection (defined in Methods). RBD sites are labeled by sub-panel of zoomed-in structural views. (G) RBD residue K378, most mutations to which mediate CR3022 escape, forms polar contacts with CR3022 residues $D54_{HC}$ and $E56_{HC}$. (H) RBD residue S383, where mutations to bulky charged or hydrophobic residues escape CR3022 binding, forms a polar contact with $K386_{RBD}$ which in turn coordinates $D101_{HC}$. S383 is also sterically constrained by close packing of the CR3022 CDRH3. (I) RBD residues V382 and F392, where mutations that alter side chain volume (V382 and F392) or hydrophobicity (F392) mediate CR3022 escape, pack with hydrophobic residues from the CR3022 CDRL1 and CDRL2 loops. (J) RBD residue T430, where mutations do not facilitate strong escape, forms a polar contact with $S27F_{LC}$ at the periphery of the CR3022:RBD interface.

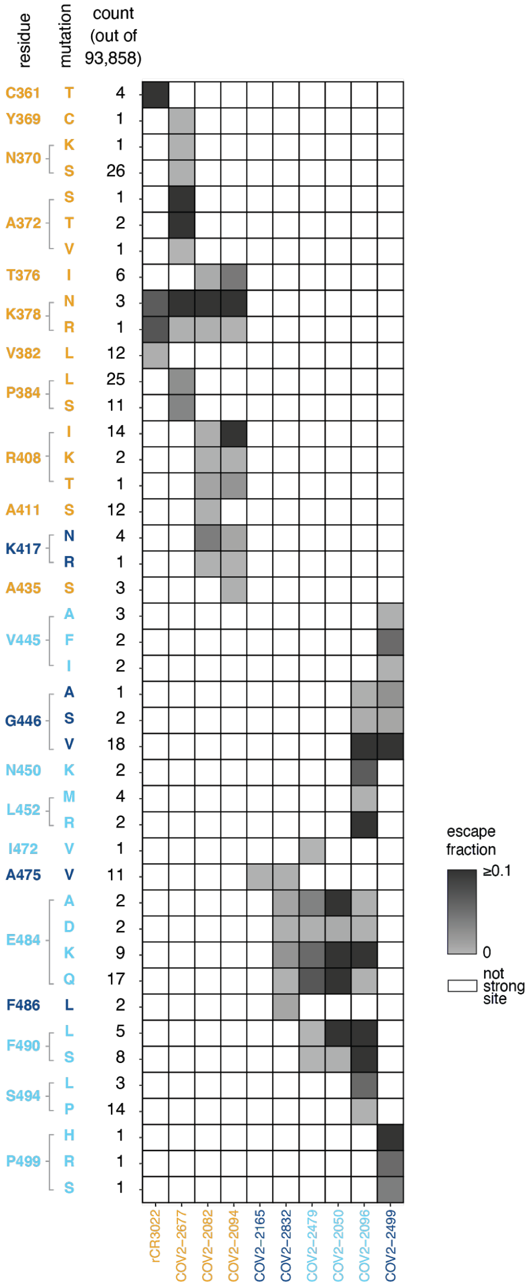

Figure S4. Variation at sites of antibody escape among currently circulating SARS-CoV-2 viruses, related to Figure 5. Table shows all RBD mutations sampled among sequences in GISAID as of 6 September 2020 at sites of escape from at least one antibody. Cells are colored by escape fraction of the individual circulating mutant for each antibody: white cells indicate sites that are not sites of escape from an antibody; for sites of escape, per-mutation escape fraction is colored from light to dark gray, with any mutation conferring >0.1 escape fraction colored equally dark. Sites are in orange for the core RBD, light blue for the RBM, and dark blue for ACE2 contact residues. Antibodies are colored according to where the majority of their sites of escape fall. These per-mutation counts are collapsed into the site-wise table presented in Figure 5A.

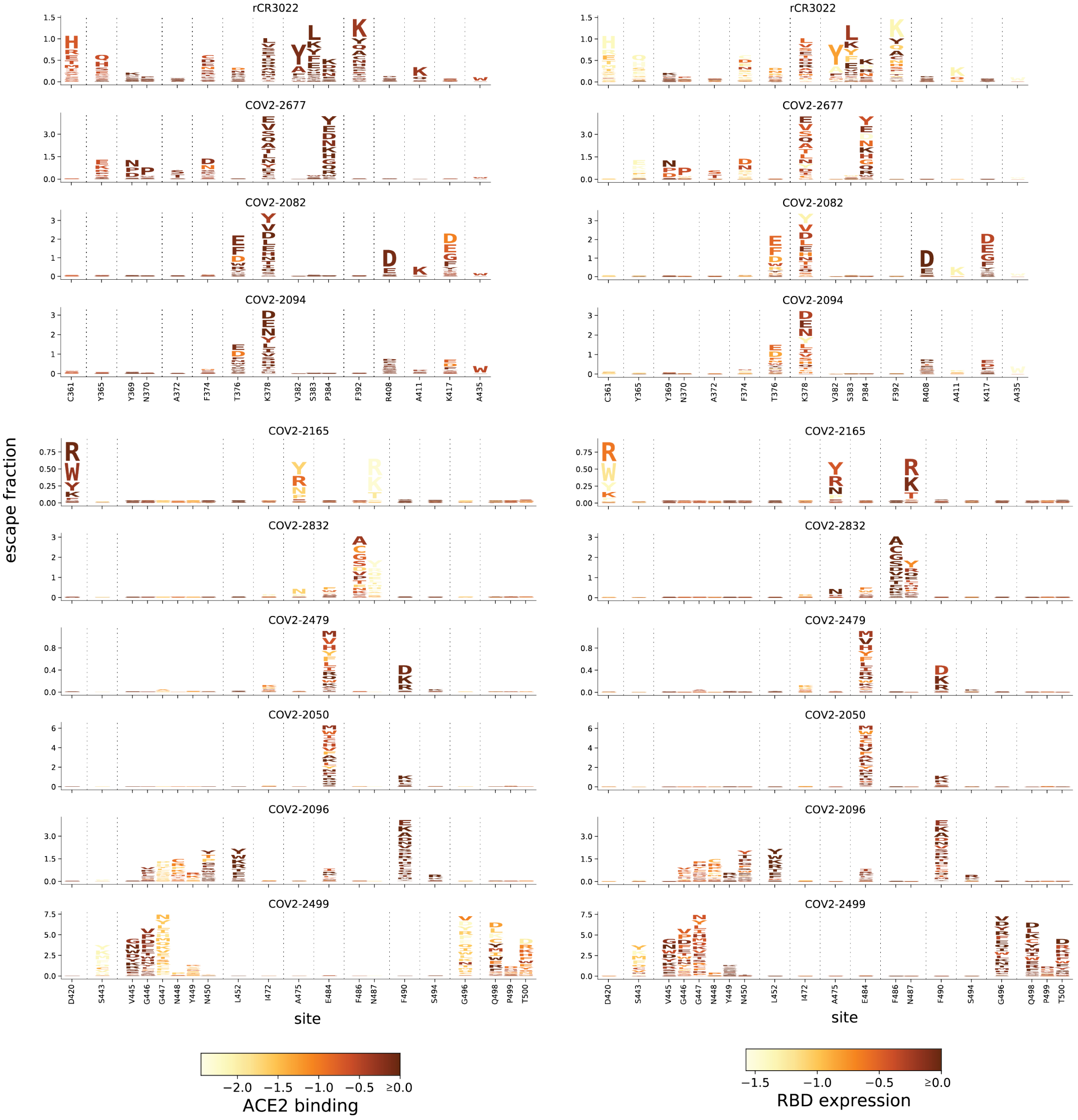

Figure S5. Logo plots of antibody escape accounting for mutation effects on ACE2-binding affinity and RBD folding, related to Figure 5. Logo plots as in Figure 2C. Mutations are colored according to their effects on ACE2-binding affinity (left) or RBD folding and expression (right), as measured previously (Starr et al., 2020). Some mutations annotated as escape in our main display impair ACE2 binding or RBD folding, which may limit their fitness in the context of virus particles.

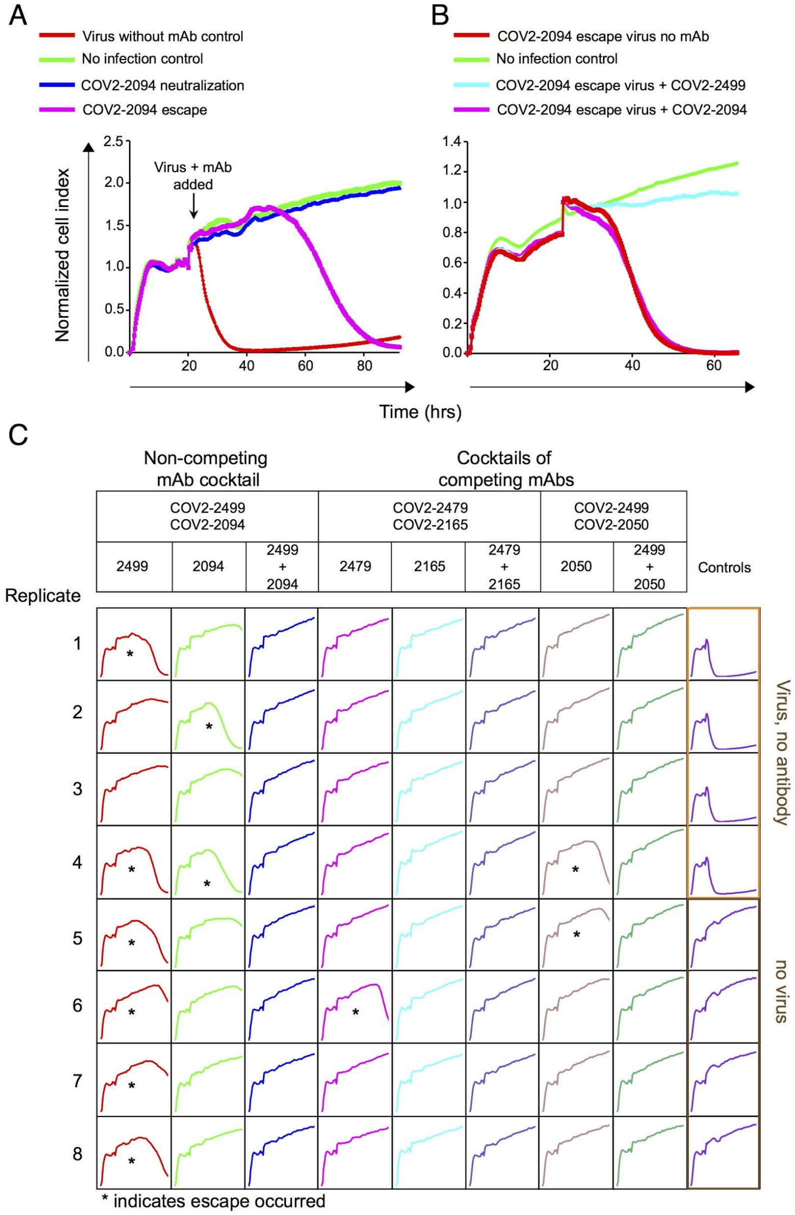

D

	Cocktail 1	
	**2499**	**2094**
**2499**	2%	89%
**2094**	78%	0%

	Cocktail 2	
	**2479**	**2165**
**2479**	0%	1%
**2165**	13%	1%

	Cocktail 3	
	**2499**	**2050**
**2499**	2%	36%
**2050**	0%	-1%

Figure S6. Real-time cell analysis (RTCA) to select for spike-expressing VSV viruses that escape antibody neutralization, and antibody competition for binding to RBD, related to Figure 6. (A) Representative RTCA sensograms showing virus that escaped antibody neutralization. Cytopathic effect (CPE) was monitored kinetically in Vero E6 cells inoculated with virus in the presence of a saturating concentration of antibody COV2-2094 (5 μg/mL). Escape (magenta) or lack of escape (blue) are shown. Uninfected cells (green) or cells inoculated with virus without antibody (red) serve as controls. Magenta and blue curves represent a single representative well; the red and green controls are mean of technical quadruplicates. (B) Representative RTCA sensograms validating that the virus selected by COV2-2094 in panel (A) indeed escaped COV2-2094 (magenta) but was neutralized by COV2-2499 (light blue). (C) Example sensograms from individual wells of 96-well E-plate analysis showing viruses that escaped neutralization (noted with *) by indicated antibodies. Escape in the illustrated replicates 6 and 7 for COV2-2499 was confirmed in validation neutralization assays but was not sequence-verified due to delayed CPE and not included in the counts in Figure 6. (D) Competition assays for RBD binding, with percentages showing binding of a second labeled antibody to the RBD after pre-binding with the first antibody. Values close to 0% indicate complete competition, and values close to 100% indicate lack of competition.

**Table S2. Summary of electron microscopy data collection and statistics for SARS-CoV-2 S protein in complex with human Fabs, related to Figure 4.**						
		**Structure of SARS-CoV-2 S2P$_{ecto}$ or S6P$_{ecto}$ proteins in complex with indicated Fabs**				
		**Fab COV2-2082**	**Fab COV2-2096**	**Fab COV2-2165 ***	**Fab COV2-2479**	**Fab COV2-2832**
	EMDB #:	EMD-22627	EMD-22148	EMD-21974	EMD-22628	EMD-22149
Microscope setting	Microscope	TF-20	TF-20	TF-20	TF-20	TF-20
	Voltage (kV)	200	200	200	200	200
	Detector	US-4000 CCD	US-4000 CCD	US-4000 CCD	US-4000 CCD	US-4000 CCD
	Magnification	50,000´	50,000´	50,000´	50,000´	50,000´
	Pixel size	2.18	2.18	2.18	2.18	2.18
	Exposure (e-/Å2)	30	30	25	30	30
	Defocus range (µm)	1.5 to 1.8	1.5 to 1.8	1.5 to 1.8	1.5 to 1.8	1.5 to 1.8
Data	Antigen	S6P$_{ecto}$	S2P$_{ecto}$	S2P$_{ecto}$	S6P$_{ecto}$	S2P$_{ecto}$
	Micrographs, #	237	562	83	331	514
	Particles, #	972	19,728	3,705	81,758	7,773
	Particles #, after 2D	673	18,202	1,868	76,431	3,778
	Final particles, #	663	12,132	1,057	18,535	3,424
	Symmetry	C1	C1	C1	C1	C1
Model docking	CoV-2-S CC	PDB: 6VYB 0.895	PDB: 6VYB 0.836	PDB: 6VYB 0.828	PDB: 6VYB 0.900	PDB: 6VYB 0.8952
	Fab (PDB: 12E8) CC	0.91	0.916	0.905	0.91	0.913

*Previously reported (Zost *et al*., 2020a**)**
